# An epifluidic electronic patch with spiking sweat clearance for event-driven perspiration monitoring

**DOI:** 10.1038/s41467-022-34442-y

**Published:** 2022-11-07

**Authors:** Sangha Kim, Seongjin Park, Jina Choi, Wonseop Hwang, Sunho Kim, In-Suk Choi, Hyunjung Yi, Rhokyun Kwak

**Affiliations:** 1 Department of Mechanical Convergence Engineering, Hanyang University, Seoul, 04763 Republic of Korea; 2 Post-Silicon Semiconductor Institute, Korea Institute of Science and Technology, Seoul, 02792 Republic of Korea; 3 Department of Materials Science and Engineering, Seoul National University, Seoul, 08826 Republic of Korea; 4 Department of Materials Science and Engineering, YU-KIST Institute, Yonsei University, Seoul, 03722 Republic of Korea; 5 Institute of Nano Science and Technology, Hanyang University, Seoul, 04763 Republic of Korea

**Keywords:** Materials science, Biomedical engineering, Health care

## Abstract

Sensory neurons generate spike patterns upon receiving external stimuli and encode key information to the spike patterns, enabling energy-efficient external information processing. Herein, we report an epifluidic electronic patch with spiking sweat clearance using a sensor containing a vertical sweat-collecting channel for event-driven, energy-efficient, long-term wireless monitoring of epidermal perspiration dynamics. Our sweat sensor contains nanomesh electrodes on its inner wall of the channel and unique sweat-clearing structures. During perspiration, repeated filling and abrupt emptying of the vertical sweat-collecting channel generate electrical spike patterns with the sweat rate and ionic conductivity proportional to the spike frequency and amplitude over a wide dynamic range and long time (> 8 h). With such ‘spiking’ sweat clearance and corresponding electronic spike patterns, the epifluidic wireless patch successfully decodes epidermal perspiration dynamics in an event-driven manner at different skin locations during exercise, consuming less than 0.6% of the energy required for continuous data transmission. Our patch could integrate various on-skin sensors and emerging edge computing technologies for energy-efficient, intelligent digital healthcare.

## Abstract (web summary)

Sensory neurons convert external stimuli into spike signals, enabling energy-efficient information processing. Here, Kwak et al. present a sensory neuron-inspired epifluidic wireless patch and demonstrate spike-based energy-efficient sweat monitoring.

## Introduction

Monitoring epidermal perspiration has attracted increasing interest due to its great potential in fitness and medical applications^1–11^. Measuring sweat rate can help people maintain appropriate hydration during physical activity^12^. It could also enable one to recognize pre-symptoms of acute physiological problems such as heart attacks and hypoglycemic shock^13^. Notably, up to 84% of people with diabetes experience excessive sweating due to nocturnal hypoglycemia^14^. Chemical information from sweat has been shown to correlate with blood composition, and obtaining that information through sweat is a promising non-invasive way to monitor health conditions^15,16^. A significant advantage of non-invasive monitoring is that it can be applied for a long time and can provide an attractive way to obtain information important for treating chronic diseases and preventing life-threatening events^13,17^.

For long-duration monitoring of epidermal perspiration, two significant improvements are needed in sweat-sensing platforms: increased operating time and design of energy-efficient wireless epidermal patches. First, the limited operating time of existing systems usually stems from the finite volume of the sweat sensors and the consequent mixing between previously collected sweat and freshly secreted sweat during the analysis^18–20^. Various approaches have been reported to address this issue, including electrochemical measurements and microfluidic systems^21,22^. When a channel or adsorption pad is used to collect sweat, it must be replaced after it is filled because the sensing electrodes cannot effectively sense newly secreted sweat if they are already full of old sweat^15,23–31^. Increasing the volume of the sweat-collecting channel could increase the operation time. Still, such a scale-up increases the channel’s hydrodynamic resistance, which inhibits passive sweat collection under natural perspiration pressure (~3 kPa), and increases the sensor size, which could make the sensor less comfortable on the skin. Very recently, a miniaturized thermal flowmeter was used to measure sweat rate^25^. Because it measured the thermal flow associated with excreted sweat, the tool could measure the sweat rate for a long time using a modestly sized sweat channel. However, the thermal approach cannot simultaneously measure sweat rate and ionic conductivity using a single device.

**Fig. 1:**
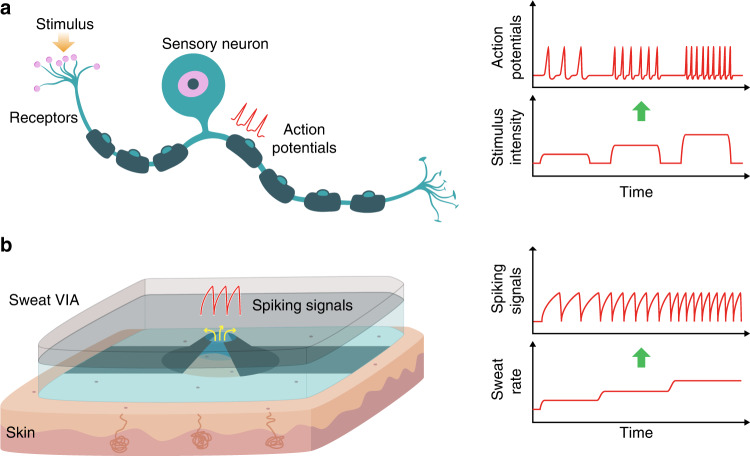
Schematic illustration of spike encoding of external stimuli of a biological sensory neuron and a sweat VIA sensor. **a** A biological sensory neuron that generates spike patterns upon receiving external stimuli through receptors with the spike frequency proportional to the stimuli intensity. **b** A sweat VIA sensor that generates spike patterns upon exposure to perspiration with the spike frequency proportional to the sweat rate, which enables event-driven, energy-efficient perspiration monitoring.

The second significant improvement that is needed involves energy consumption and data storage required to secure long-term, wireless sensor functionality^32,33^. Although wireless monitoring is highly desirable in terms of user convenience and the facile application required for digital healthcare, wireless transmission of real-time, continuous data incurs significant energy costs and produces a large amount of often redundant data. Therefore, it is desirable to devise an event-driven wireless monitoring scheme that will transmit data only when relevant events occur while providing the most essential features of the measured health marker–related signals. One way to materialize event-driven monitoring is to encode key information on external stimuli into spike patterns like sensory neurons^34–36^. Upon external stimulation, sensory neurons generate spikes at a frequency proportional to the intensity of the stimulus, as illustrated in Fig. 1a. Such event-driven spike-based signal processing of the neuron is energy-efficient and resistant to noise and improves real-time, event-driven sensory processing^32^. To the best of our knowledge, although neuron-inspired spike encoding of bio-signals has been reported with additional sensors and circuitry^37,38^, an approach that can directly encode perspiration into spikes and enable event-driven, energy-efficient, wireless monitoring of epidermal perspiration dynamics has been elusive.

Herein, we report an epifluidic electronic patch with “spiking” sweat clearance and demonstrate its ability to enable event-driven, wireless monitoring of epidermal perspiration dynamics. To this end, we first developed a sensor containing an open, truncated cone-shaped vertical sweat channel with a pair of nanomesh electrodes on its inner wall and a sweat-clearing structure at the channel top to empty the sweat in the sensor abruptly. With an analogy to vertical interconnect access (VIA) of the printed circuit board (PCB), we named this device a sweat VIA sensor. Emptying the sweat VIA sensor produces a sharp change in admittance and allows newly secreted sweat to be collected during perspiration without mixing with old sweat, which enables the sensor to generate a series of spike signals whose frequency and amplitude are proportional to the sweat rate and ionic conductivity, respectively. We further developed the sweat VIA-based epifluidic wireless patch that transmits peak data upon spiking events induced by perspiration, and demonstrated its ability to decode epidermal perspiration in an event-driven manner at different skin locations during heavy exercise for 100 min. Our sweat VIA-based epifluidic patch could be integrated with other types of on-skin sensors and emerging edge computing technologies to enable energy-efficient, intelligent digital healthcare.

## Results

**Fig. 2:**
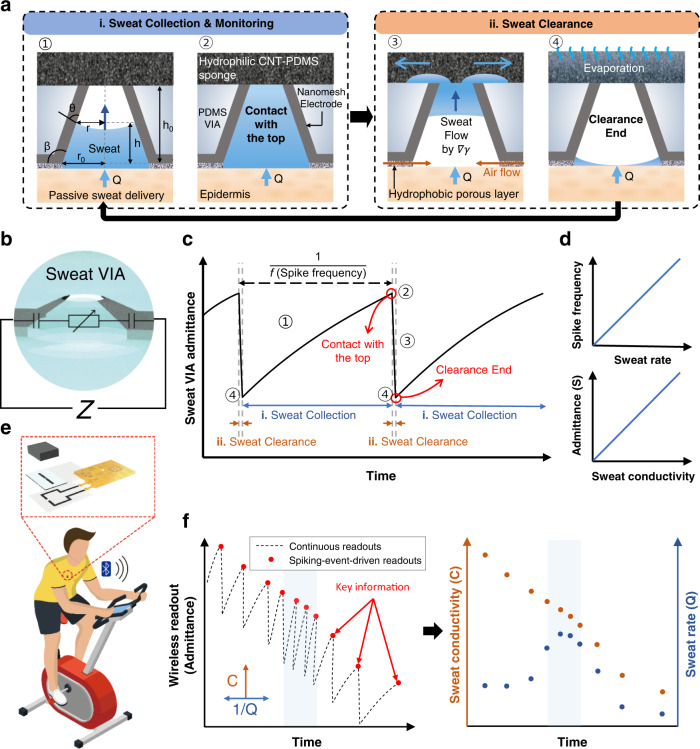
Structures and operating principles of the sweat VIA sensor and event-driven wireless monitoring of epidermal perspiration. Schematic diagrams of **a** the structures and operating principles of the sweat VIA sensor and **b** its equivalent circuit. **c** Example of a spike admittance curve produced by the sweat VIA sensor during the collection and clearance of sweat. **d** Schematic of the sweat rate versus spike frequency and sweat conductivity versus peak admittance of the spike signal. **e** The sweat VIA-based epifluidic wireless patch that wirelessly transmits measured data to a mobile phone via BLE. **f** Illustration of spiking-event-driven data transmission containing key information about sweat rate and ionic conductivity. The spike frequency and amplitude encode the sweat rate and ionic conductivity, respectively, with the spiking-event-driven readout requiring many fewer wireless data transmissions than a continuous readout.

Figure 1a schematically illustrates the biological sensory neuron that generates spike patterns upon receiving external stimuli through receptors with the spike frequency proportional to the stimuli intensity. Similarly, the sweat VIA sensor generates spike patterns upon exposure to perspiration, with the spike frequency proportional to the perspiration rate (Fig. 1b). Figure 2a illustrates the structure and operating principle of the sweat VIA sensor in detail. The sensor was designed to collect epidermal sweat into a vertically oriented VIA channel under the natural pressure of perspiration (<3 kPa). The collected sweat flows along the VIA channel until it reaches the top hydrophilic layer, which facilely and abruptly absorbs the sweat and empties the VIA channel. To achieve passive collection and facile clearance of sweat under the pressure of natural perspiration, we designed the VIA channel as a very short, open, truncated cone with a significantly low hydrodynamic resistance. The truncated cone-shaped VIA channel also collects sweat enough through its wide bottom and minimizes natural evaporation through its narrow, open top.

When the VIA channel is filled, the sweat is effectively absorbed by a super-hydrophilic sponge on its top surface, which empties the channel and enables the sensor to characterize newly produced sweat (Fig. 2a). This filling and the emptying process is repeated as long as perspiration continues, so the sensor’s operation time is not limited by VIA channel volume. The sponge has a large surface-to-volume ratio to facilitate evaporation of the absorbed sweat, which allows a large quantity of newly produced sweat to be absorbed during the long-term operation of the sweat VIA sensor (Fig. 2a). Because the volume of air provided must be the same as the volume of cleared sweat to effectively empty the VIA channel (analogous to an air venting passage during injection molding), a hydrophobic porous layer is attached to the bottom of the channel to facilitate airflow into the channel during sweat clearance. This porous layer also helps to cut the liquid bridge of sweat flow during sweat clearance to cause an abrupt clearance of the sweat.

The collection and clearance of sweat are electrically monitored by a pair of nanomesh electrodes placed on the sidewall of the VIA channel to measure the admittance (S) of sweat, as illustrated in Fig. 2b–d. The equivalent circuit of this sensor consists of a series connection of two electrical double-layer-like interfacial capacitors and a resistor whose resistance value is determined by the bulk resistance of sweat^39^. To determine the sweat’s ionic concentration, the admittance of sweat is measured in a frequency range higher than the characteristic frequency of the equivalent RC circuit, defined as 1/τ (=1/2$\pi$RC). Above that characteristic frequency, the resistive component is dominant, and the measured admittance can be correlated with the ionic concentration of sweat, as demonstrated below.

A schematic admittance curve produced by the sweat VIA sensor during the collection and clearance of sweat is illustrated in Fig. 2c. The characteristic features corresponding to the schematic shown in Fig. 2a are also indicated. When the sweat VIA sensor is emptied (4), the admittance of the sweat VIA channel is low. As the sweat flows into the VIA channel, the admittance increases (1) until the sweat reaches the top sponge layer (2). Once the sweat meets the top sponge, it is cleared, and the admittance of the channel begins to decrease (3), reaching a minimum level upon completion of the clearance (4). It is noted that clearing the sweat from the channel takes less time than collecting it. Therefore, a cycle of filling and abrupt ‘spiking’ emptying the VIA channel produces a spike in the signal, as illustrated in Fig. 2c. The sweat VIA sensor produces a series of spike signals as perspiration continues. The measured spike frequency combined with the designated VIA channel volume can be used to determine the sweat rate, with higher sweat rates associated with higher spike frequencies. Moreover, because the admittance of the filled VIA is proportional to the ionic conductivity of the sweat, it is proportional to the ion concentration, as schematically illustrated in Fig. 2d (see Supplementary Information for detailed calculations).

Figure 2e illustrates our sweat VIA-based epifluidic patch, which measures the admittance of the sweat VIA sensor. It wirelessly transmits the relevant data to a mobile device using the Bluetooth Low Energy (BLE) protocol. Because the spike frequency and amplitude encode the sweat rate and ionic conductivity, the data can be wirelessly transmitted to the mobile device only when spikes are generated, as depicted in Fig. 2f. In this manner, key information on epidermal perspiration dynamics (sweat rate and ionic conductivity) can be fully decoded in a small number of wireless data transmissions, which minimizes power consumption.

**Fig. 3:**
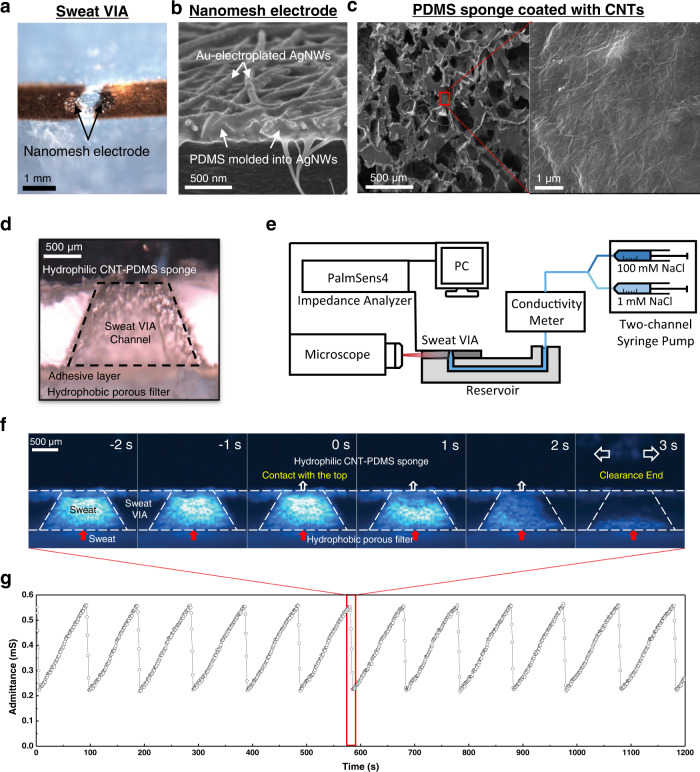
Characteristics of the sweat VIA sensor. **a** Photograph of the VIA channel and nanomesh electrode. Scanning electron microscope images of **b** the nanomesh electrode and **c** CNT-PDMS sponge. **d** Side-view optical image of the assembled sweat VIA sensor. **e** Experimental setup for visualizing sweat flow in the sweat VIA sensor and measuring admittance. **f** Fluorescence micrographs showing clearance of the sweat solution as it contacted the CNT-PDMS sponge. **g** Measured admittance curve from the sweat VIA sensor during the continuous flow of a 92.7 mM NaCl solution (10,350 μS/cm) at a flow rate of 0.5 μL/min. Solution clearance occurred at each point at which the admittance suddenly decreased (in the red box for one case).

Figure 3a shows an optical image of the VIA channel, which is made of polydimethylsiloxane (PDMS) and nanomesh electrodes. The dimensions of the VIA channel were set considering both passive collections of sweat under the pressure of natural perspiration (~3 kPa) and the fabrication processes. Various channel geometries were tested. They all had a channel inlet radius (r_0_) of 0.75 mm and a wall angle (β) of 116.7°. Still, they had different channel heights (h_0_) ranging from 0.35 to 1 mm and correspondingly different top outlet radii ranging from 0.625 to 0.25 mm, respectively. For a channel height of 1 mm, the Darcy–Weisbach equation showed that the hydrodynamic resistance under an extremely high flow rate of 10 μL/min (3000 μL/h/cm^2^) was ~6.5 Pa, much lower than the perspiration pressure (the sweat rate during heavy exercise is about 300 μL/h/cm^2,^^15^). The pair of nanomesh electrodes were fabricated on the sidewall of the VIA channel using a hydrogel-templated molding transfer method previously reported by our group with slight modifications (see the Methods section and Supplementary Fig. S1a)^40^. Briefly, conductive nanomesh electrodes of silver nanowires (AgNWs) and carbon nanotubes (CNTs) were formed on a hydrogel surface, and a PDMS solution was cured on that surface. During the curing process, the PDMS solution slightly penetrated the AgNW networks, as shown in the scanning electron micrograph of the nanomesh (Fig. 3b), and provided a stable interface between the PDMS body and the nanomesh electrode. In that way, the AgNW/CNT electrodes were transferred from the hydrogel surface to the PDMS body when the cured PDMS was peeled off to produce the VIA channel. The AgNW/CNT electrode was further electroplated with inert Au (Ep-Au) to improve its biocompatibility^41^. Elemental mapping analysis of the nanomesh electrodes confirmed that the AgNW surface was successfully coated with the Au layer (Supplementary Fig. S2). The Ep-Au also slightly reduced the impedance of the nanomesh electrodes at low frequencies (Supplementary Fig. S3).

The sweat clearance layer was fabricated using a microporous PDMS sponge. The sponge was fabricated by curing a PDMS solution on a sugar template with an average particle size of ~300 μm and then dissolving the sugar template. The surface of the PDMS sponge was then subjected to a plasma treatment, after which it was coated with CNTs (see the Methods section and Supplementary Fig. S1b). The scanning electron micrographs of the resulting CNT-PDMS sponge confirm that CNT networks formed on the surface of the microporous PDMS sponge (Fig. 3c). The nanonetwork structure of the CNTs, in combination with the microporous PDMS sponge, allows for both rapid absorption of the sweat and a high evaporation rate (Supplementary Fig. S4–5). In our tests, the CNT-PDMS sponge absorbed an aqueous solution at the rate of 2.5 μL per mg of sponge, and the liquid evaporated from the sponge rapidly (e.g., 1.3 μL/min per cm^2^ of sponge at 25.5 °C and 20% humidity, Supplementary Fig. S5). Considering that the sweat rate on the skin during mild/heavy exercise is 10–1000 μL/h/cm^2,^^42^, the large absorption and evaporation capacity of the CNT-PDMS sponge are suitable for effective clearance of sweat during long-term operation of the sweat VIA sensor. As confirmed through our on-body study, the cumulative evaporation volumes were greater than the cumulative sweat loss. An optical image of the completed sweat VIA sensor assembled with the top hydrophilic sponge and bottom hydrophobic layer is shown in Fig. 3d.

To characterize the sweat clearance dynamics of the sweat VIA sensor and monitor the electrical admittance of the VIA channel in the presence of a sweat-mimicking solution, we set up an inverted fluorescence microscope, an impedance analyzer, and a microfluidic system, as shown in Fig. 3e. Solutions of 1 mM NaCl and 100 mM NaCl were mixed at different flow rates as sweat-mimicking solutions of various ionic concentrations. Figure 3f shows a series of fluorescence micrographs of the sweat VIA sensor taken as snapshots during the continuous flow of the NaCl solution. The solution in the VIA channel was cleared within 3 s of coming into contact with the top CNT-PDMS layer. The impedance of the sweat was measured using AC voltage at a frequency of 50 kHz and an amplitude of 30 mV. The measurement frequency of 50 kHz was determined based on an EIS curve obtained using the sweat VIA sensor, where the resistive component (and therefore the solution resistance) was the dominant determinant of the impedance (frequency >~1 kHz) (Supplementary Fig. S6). A representative admittance curve for the sweat VIA sensor at a continuous flow of 0.5 μL/min and a NaCl solution at a concentration of 92.7 mM (10,350 μS/cm) is shown in Fig. 3g. A series of spikes are clearly visible in the admittance curves.

**Fig. 4:**
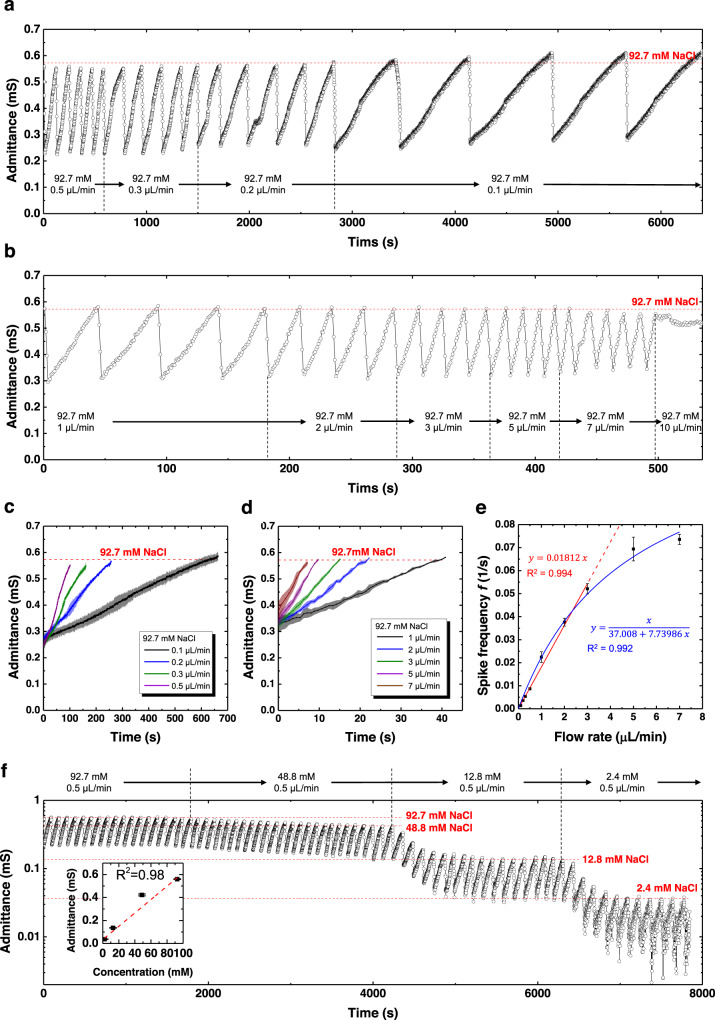
Encoding the flow rate and ionic conductivity into admittance spikes using the sweat VIA sensor. Continuous monitoring of the electrical admittance when the simulated sweat (92.7 mM NaCl solution, which has a conductivity of 10,350 μS/cm) experiences a change of flow rate **a** from 0.1 to 0.5 μL/min and **b** from 1 to 10 μL/min. The average spiking signals of the admittance curve at each flow rate of **c** 0.1–0.5 μL/min and **d** 1–10 μL/min. **e**. Dependence of spike frequency on the flow rate calculated from the admittance curves in **a** and **b**. The data points for flow rates of 0.1–3 μL/min were well fit by a straight line (red line), but all the data points were better fit by a nonlinear curve considering a non-negligible sweat clearance time (blue line). The detailed derivation of the fitting equations is given in the Supplementary Information. **f** Continuous monitoring of the admittance of the sweat VIA sensor with aqueous solutions of NaCl at concentrations of 2.4, 12.8, 48.8, and 92.7 mM (273; 1,737; 4,945; and 10,350 μS/cm), each at a flow rate of 0.5 μL/min, and the resulting encoding of ionic concentration into the admittance peak amplitude.

Next, the capability of the sweat VIA sensor to produce spikes in admittance and encode the sweat rate and ionic conductivity into spike frequency and amplitude was examined (Fig. 4). The sweat VIA sensor was fed an aqueous NaCl solution at various flow rates from 0.1 to 10 μL/min, and the admittance was monitored continuously. Plots of admittance versus time for flow rates of 0.1–0.5 μL/min and 1–10 μL/min are presented in Fig. 4a, b, respectively. The spike frequency decreased (increased) as the flow rate decreased (increased). The sweat VIA sensor generated spikes in admittance for flow rates of up to 7 μL/min, but the admittance remained at or near its peak value for a flow rate of 10 μL/min, presumably due to the continuous filling of the channel with sweat as the CNT-PDMS sponge was clearing it. The averages of the spiking portions of the admittance curve obtained from each flow rate are presented in Fig. 4c, d. Each flow rate generated a different admittance curve. The dependence of spike frequency on flow rate is summarized in Fig. 4e. The spike frequency increased markedly and linearly with a flow rate up to 3 μL/min and then increased more slowly. This reduction in the rate of increase in spike frequency can be ascribed to the sweat clearance rate, which started to be not fast enough than the sweat collection rate; the rate of growth can be calibrated by correcting the fitting equation with a non-negligible constant clearance time (blue line in Fig. 4e). This wide dynamic range of flow rates, from 0.1 to 7 μL/min, allowed us to monitor perspiration in conditions from mild exercise such as walking (10–100 μL/h/cm^2^) to heavy exercise (100–1000 μL/h/cm^2,^^15^). Also, the dynamic range of the sweat rate can be shifted by adjusting the geometry of the VIA channel; for example, it could be shifted to lower values by increasing the area of the opening at the channel bottom and decreasing the height to secure a smaller channel volume.

We next examined the dependence of the spike in the signal on ionic conductivity. The ionic conductivity was continuously controlled using two NaCl solutions with different ionic concentrations (1 and 100 mM) and by adjusting the flow rate of each solution. The measured admittance profiles using ionic concentrations of 2.4–92.7 mM (273–10,350 μS/cm) are presented in Fig. 4f. Here, the highest admittance spikes for each ionic concentration show discrete levels and correlate with the ionic concentration of the solution. Specifically, the lower the ionic conductivity, the lower the peak admittance of each spike, with each peak admittance corresponding to a VIA channel filled with sweat. Because the volume of the filled VIA channel was fixed, the admittance can be correlated directly with the conductivity of the solution (Supplementary Discussion). When non-conductive components such as urea, serine, and glycine, which are present in human sweat at high concentrations^43,44^ were added to the testing solution, neither the peak admittance nor the frequency of spike signals was changed (Supplementary Figs. S7, S8). Thus, the spike frequency and the highest value of the admittance spike can encode, respectively, the sweating rate and the sweat’s ionic conductivity.

Lastly, we tested the long-term stability and reproducibility of the sweat VIA sensor (Supplementary Discussion). Through 24 h long-term tests, we clarified that the relative magnitude between the evaporation rate of the sponge and the sweat rate affected the operation time of the sweat VIA device (Fig. S9). When the evaporation rate was higher than the sweat rate, the sweat VIA device operated for 24 h and monitored a total sweat solution volume of 720 μL. In the opposite condition, however, the sweat VIA device operated for ~7.6 h until the residual sweat after evaporation inside the sponge reached ~ 38% of the absorption capacity of the sponge. Next, three different devices operated for 8 h at a flow rate of 0.5 μL/min, which showed similar levels of admittance and the period of spike signals with only slight variations, demonstrating the high reproducibility of our device (Fig. S10).

**Fig. 5:**
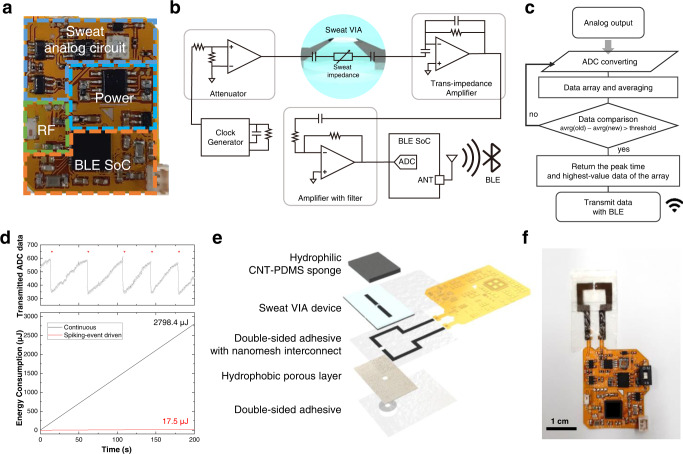
A sweat VIA-based epifluidic wireless patch. **a** Photograph of the flexible printed circuit board–based wireless sensor platform for monitoring epidermal perspiration dynamics using the sweat VIA sensor. **b** Schematic diagram of the sweat VIA-based wireless sensor platform. **c** System-level block algorithm for the sweat VIA sensor enabling spiking-event-driven wireless data transmission. **d** Comparison of the energy consumption of wireless data transmission in a continuous manner with that in a spiking-event-driven manner. **e** Exploded-view illustration of the sweat VIA-based epifluidic wireless patch. **f** Photograph of the assembled sweat VIA-based epifluidic patch. For clarity, the CNT-PDMS sponge and battery are not shown here.

Figure 5a shows the flexible printed circuit board (FPCB)-based wireless sensor platform used for event-driven wireless monitoring of sweat rate and ionic conductivity. The wireless sensor system consists of a sweat VIA sensing module, a BLE SoC (system on a chip) for control and wireless communication, and a power management system. Its operational process is illustrated in Fig. 5b. To measure the admittance of the sweat VIA sensor, the sensing module uses a clock generator and trans-impedance amplifier (TIA). An AC voltage of 70 kHz and 30 mV is generated using the clock generator and an attenuation circuit: the AC voltage is applied through the sweat VIA sensor, and the admittance is measured using the TIA. The output signal of the TIA is connected to the BLE SoC via an analog-to-digital converter (ADC): the ADC output data are wirelessly transmitted to the user’s smartphone through BLE, and then the wireless readout data are converted to admittance values using a calibration curve. The wireless sensor platform did not show a noticeable self-heating effect even when continuously operated for longer than 8 h, suggesting its suitability for an on-skin platform (Supplementary Fig. S11).

Because the sweat VIA sensor is designed to monitor the wearer’s sweat rate and ionic conductivity, it only needs to send admittance data when spikes are generated, and the necessary spike frequency and amplitude information can be calculated from those data. We, therefore, devised an algorithm, shown in Fig. 5c, to have the system send an admittance value only when a spike is generated. In this scheme, the average values of two consecutive arrays of old and new data arranged in chronological order are compared to locate the spiking point—where the difference between the average values is larger than the threshold value set based on the sweat clearance—and only then are the spiking data transmitted. In this way, the number of data transfers can be minimized. For example, Fig. 5d compares the energy required for continuous and spiking-event-driven wireless data transmissions. Over 200 s, the spiking-event-driven monitoring requires only 5 data transfers, whereas continuous monitoring requires 800 data transfers. In this case, the spiking-event-driven transmission of admittance data would consume 17.5 μJ of energy (Supplementary Fig. S12), only 0.63% of the 2798.4 μJ of energy required by continuous data transmission (Fig. 5d). Therefore, compared with continuous monitoring, event-driven monitoring can significantly reduce the energy required for wireless data transfer and still provide relevant information about epidermal perspiration.

**Fig. 6:**
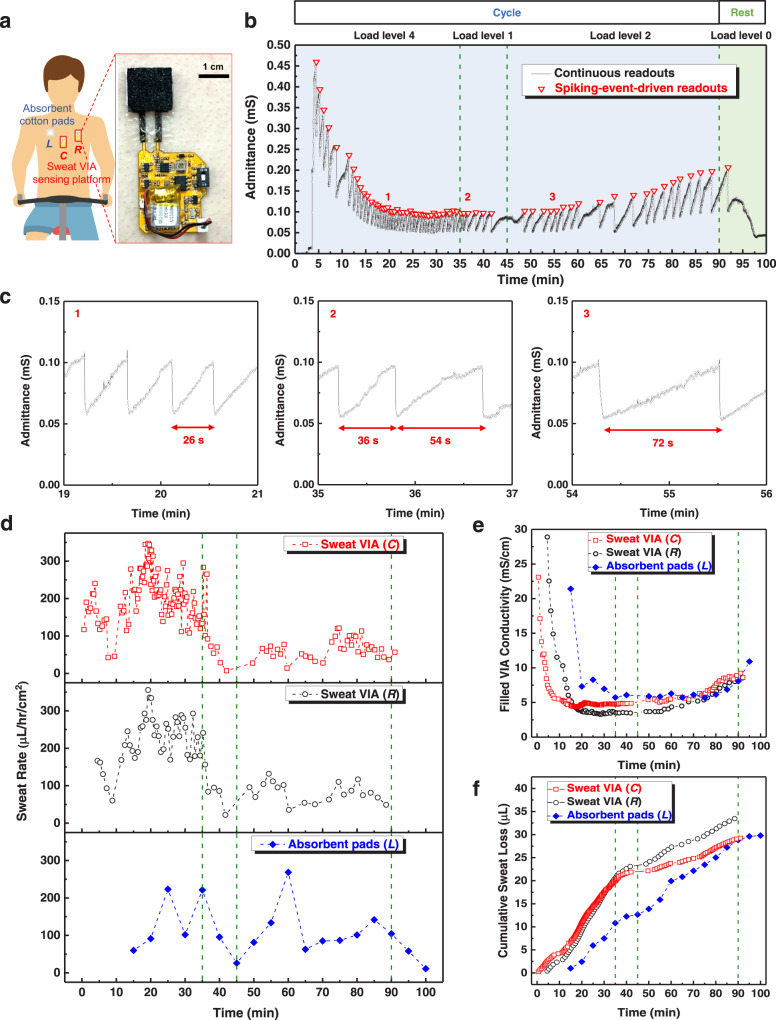
On-body study of event-driven wireless monitoring of epidermal perspiration. **a** Schematic and photographic images of the on-body study using the sweat VIA-based epifluidic wireless patches (*C*, *R*) and absorbent cotton pads (*L*) attached to the indicated chest skin areas. *C*: center, *R*: right, *L*: left area of the chest skin. **b** Admittance values of the sweat VIA sensor calculated using wireless readouts transmitted in continuous (black line) and spiking-event-driven (red inverted triangle) manners during the entire exercise sequence of 100 min with various cycling load levels, with the higher number indicating a harder exercise load. **c** Enlarged graphs of the continuous admittance data shown in **b** obtained under different exercise load levels. **d** Sweat rates and **e** conductivity levels were obtained using the spiking-event-driven wireless readouts from each epifluidic patch and the laboratory analysis of the sweat collected manually using absorbent cotton pads during the exercise sequence shown in **a**. **f** Measured volumes of cumulative sweat loss through the sweat VIA-based epifluidic patch and absorbent pads. In panels, **b**–**f**, the dashed green lines mark when the cycling load level changed.

An exploded-view illustration of the epifluidic electronic patch is provided in Fig. 5e. The sweat VIA sensor and FPCB-based wireless sensor platform are electrically connected using Au wires that specifically connect the nanomesh electrodes with the contact pads of the FPCB. A double-sided skin-safe adhesive layer based on the transparent biomedical film Tegaderm^TM^ (3M) was used to attach the sweat VIA sensor to the skin. A hole with a diameter of 5 mm was formed in this layer and aligned with the VIA channel to allow the effective collection of epidermal sweat. A photograph of the assembled sweat VIA-based epifluidic patch is provided in Fig. 5f. In that picture, the CNT-PDMS sponge and the battery are not shown to provide a more transparent presentation of the assembled device structure. A photograph of the full epifluidic patch with the CNT-PDMS sponge and connected battery is provided in Fig. 6a.

We evaluated the event-driven wireless monitoring of epidermal perspiration dynamics using the sweat VIA-based epifluidic patch in trials of male human participants performing physical activity. Two patches were attached to the chest skin area of each volunteer at the positions denoted as *C* (center) and *R* (right) in Fig. 6a, and the sensor data were wirelessly transmitted to a smartphone. Both continuously collected and spiking-event-driven data were wirelessly transmitted for comparison. To compare the data from the epifluidic patches with those from manually collected sweat, absorbent cotton pads were attached to the chest skin at the position marked as *L* (left) in Fig. 6a, symmetrically across from the position of sensing platform *R*. The absorbent cotton pads were replaced every 5 min, whereas the epifluidic patches were used continuously for the whole exercise sequence. The participants performed stationary cycling for 90 min with varying exercise load levels, followed by a rest of 10 min. The admittance values calculated from the wireless readouts obtained from the sweat VIA sensor (Supplementary Fig. S13) throughout the 100 min sequence are presented in Fig. 6b.

Characteristic spike peaks of the sweat VIA-based patch were observed in the continuous wireless readout data (black curve), and the spiking-event-driven wireless readouts (indicated as red inverted triangles) correlated well with the points for spike peaks. The frequency of the spiking events correlated with the cycling load level such that higher cycling loads produced higher spike frequencies (Fig. 6b, c). Sweat rates were calculated using the wireless readout data for the spiking-driven events shown in Fig. 6b, calibration curves for each wireless sensor system (Supplementary Fig. S13), and the volume of the VIA channel. The sweat rates from each patch were compared with the sweat rate obtained by analyzing the sweat manually collected using the absorbent cotton pads (see the Methods). Those results are summarized in Fig. 6d.

Interestingly, the epifluidic patch at the center chest area (*C*) produced sweat spikes earlier than the patch at the outer area (*R*), suggesting that epidermal sweat was secreted first from the center of the chest skin and then at the outer area (*R*). These results also confirm the correlation between load level and the sweat rates obtained from the epifluidic patches and the absorbent cotton pads. However, the absolute values and the timing differed slightly, presumably due to errors in the manual collection of sweat. The results also suggest that the event-driven wireless monitoring scheme of the sweat VIA-based epifluidic patch provided a larger number of fine data points for sweat rate in a manner significantly more convenient than manual collection. More importantly, the spiking-event-driven monitoring scheme successfully decoded the epidermal perspiration using only about 0.57% (*C*) and 0.27% (*R*) of the number of wireless data transmissions used for continuous monitoring.

The sweat conductivity values were calculated using the highest admittance value of each spike and the calibration curve for each patch (Supplementary Fig. S14). The conductivity value of the manually collected sweat was obtained using laboratory equipment (see the Methods and Supplementary Fig. S15). These conductivity results are summarized and compared in Fig. 6e. The ionic conductivity values were relatively high at the beginning of the cycling for all three cases and then gradually decreased, presumably due to the dilution of sweat as perspiration continued. The conductivity did change somewhat with a change in cycling load, though to a lesser degree than the initial decrease in conductivity. These results demonstrate that the sweat VIA-based epifluidic wireless patch can simultaneously measure the sweat rate and conductivity using a single device and in an event-driven manner. The total sweat loss is shown in Fig. 6f. A large amount of sweat, 30 μL, was monitored electrically using a single device. Compared with the sweat-clearing capability of the sweat VIA with the sweat loss during the on-body test, our sensor system could monitor the sweat dynamics for longer than 24 h (Supplementary Fig. S5). All these results highlight the potential of our epifluidic patch for long-term, wireless, and energy-efficient monitoring of sweat rate and conductivity.

We developed an epifluidic wireless patch with spiking sweat clearance using the sweat VIA sensor that generates spike patterns upon exposure to perspiration. We demonstrated the encoding and decoding processes for using those spikes for long-term, event-driven, wireless monitoring of epidermal perspiration. The sweat VIA sensor with nanomesh sidewall electrodes and a sweat-clearing sponge layer enables cycles of filling and emptying the VIA channel. The filling-emptying process allows us to determine epidermal perspiration dynamics from spikes in the admittance signals and to monitor those signals in a long-term, energy-efficient manner despite the small volume (<1 μL) of the VIA channel. Trials with human participants demonstrated that the sweat VIA-based epifluidic wireless patch could operate for a long time and a large amount of sweat (> 30 μL) using a single device. The spiking-event-driven wireless readouts successfully decoded epidermal perspiration from different skin areas during exercise. With such an event-driven and long-duration operation, our sweat VIA sensor could be used to detect pre-symptoms of acute diseases or health conditions such as nocturnal hypoglycemic shock and heart attacks. In addition, the sweat VIA sensor could be further integrated with other types of on-skin sensors that need to be operated for long periods and be combined with emerging computing technologies to enable energy-efficient, intelligent digital healthcare.

## Methods

### Fabrication of the sweat VIA sensor with nanomesh electrodes on the channel sidewall

The sweat VIA sensor was fabricated according to the method previously reported by our group with slight modifications^40^. The fabrication procedure is also illustrated in Supplementary Fig. S1a. An agarose hydrogel (5% w/v, Bioline) solution was molded using a 3D-printed plastic master mold engraved with a truncated cone with a height of 1 mm, a bottom diameter of 1.5 mm, and a top diameter of 0.5 mm. Onto that molded hydrogel template, solutions of AgNWs (average diameter of 21 ± 3 nm, average length of 22 ± 5 μm, SG Flexio) and CNTs (average diameter of 1–2 nm, average length of 5–10 μm, Duksan Pure Chemical) were sequentially spray-coated through a pre-patterned mask. The AgNWs and CNTs formed nanonetworks on the hydrogel surface, and the surfactants and impurities in the solution were removed through the hydrogel’s pores. Then, a PDMS solution was poured onto the hydrogel surface and cured at 65 °C for 2 h. The AgNWs and CNTs were transferred to the PDMS during the curing process. The cured PDMS layer was peeled off to produce a truncated cone-shaped VIA channel with nanomesh electrodes of AgNWs and CNTs on its inner wall. The nanomesh electrodes were then electroplated with an Au solution at −0.92 V for 10 s to coat the nanomesh surface with an inert Au layer.

### Fabrication of the super-hydrophilic PDMS-CNT sponge

The procedure used to fabricate the super-hydrophilic CNT-PDMS sponges is illustrated in Supplementary Fig. S1b^45^. First, a cube of sugar with a particle size of ~300 μm was immersed in a PDMS solution with a 10:1 mass ratio of PDMS prepolymer to curing agent (Sylgard 184, Dow Corning), followed by degassing of the resulting mixture in a vacuum chamber for 2 h to remove air bubbles. The sugar cube absorbed the PDMS precursor, and the resulting composite was cured at 65 °C for 2 h and then dissolved in warm water to produce a microporous PDMS sponge. The average size of the micropores was about ~300 μm. The PDMS sponge was treated with O_2_ plasma, immediately dipped into a CNT solution (Duksan Pure Chemical, South Korea), and rinsed several times with deionized water to remove the surfactant.

### Assembly of the sweat VIA sensor

Interconnected nanomesh electrodes of AgNWs and CNTs for electrical measurements were patterned by spray coating through a patterned mask on a double-sided skin-safe adhesive containing a hole with a diameter of 1.5 mm. This adhesive was fabricated by bonding two Tegaderm^TM^ (3M) biomedical dressing films at 150 °C for 5 min. The adhesive with the patterned interconnect was attached to the sweat VIA sensor. A hydrophobic layer made of a porous filter (PVDF 0.45 μm pore syringe filter, CNW Technologies) was cut to form a hole with a diameter of 1.5 mm and attached to the adhesive-attached sweat VIA sensor, taking care to align the hole with the channel. The CNT-PDMS sponge was placed on top of the sensor.

### Characterization of the sweat VIA sensor using a microfluidic system

A two-channel syringe pump with a controllable flow rate (Q = 0.1–10 μL/min) and a sweat reservoir were set up as illustrated in Fig. 3e. NaCl solutions of various ionic concentrations from 1 to 100 mM were used to emulate sweat. The two-channel syringe pump was connected to the bottom of the sweat VIA sensor; here, one syringe was filled with a solution of 1 mM NaCl and the other with 100 mM NaCl. The admittance of the sweat VIA was measured at a frequency of 50 kHz and 0.01 V. The exact conductance of the injected solution was also measured using a microscale flow-through conductivity meter. To examine the dynamic range of the flow rate of the sweat VIA sensor, a NaCl solution with a fixed ionic concentration was applied with 10 flow rates (Q = 0.1–10 μL/min). Here, a solution of 100 mM NaCl was connected to the reservoir; however, due to the resulting diffusion, the actual ion concentration provided to the sensor was 92.7 mM (10,350 μS/cm). The dynamic range of the sensor with regard to ionic concentration was examined by testing various ionic concentrations at a fixed flow rate of 0.5 μL/min: 92.7 mM (10,350 μS/cm), 48.4 mM (4945 μS/cm), 12.8 mM (1737 μS/cm), and 2.4 mM (273 μS/cm).

### Fabrication of the FPCB-based wireless sensor platform

A flexible copper-clad laminate (DSflex-600 122512E, DOOSAN) composed of a thin film of copper/PI/copper (thickness of 12 μm each) that served as the substrate of the FPCB, electroplated copper with a thickness of 10 μm, and a cover layer (12.5-μm-thick PI and 15-μm-thick adhesive, MAH-0X-25NX, INNOX) were used to fabricate the wireless sensor system. The exposed top and bottom copper layers were plated with electroless nickel immersion gold (thickness of 3.5 μm Ni, 0.03 μm Au) to form soldering pads for component mounting. Soldering paste (LF999, KELLYSHUN) and wire (XL806, Alpha Assembly Solutions) were used to mount the necessary components onto the FPCB, including the BLE microcontroller (nRF52832, Nordic Semiconductor), amplifier (LTC2066 and LTC6081, Analog Devices), clock generator (LTC6900, Analog Devices), linear regulator (AP2112K, Diodes Incorporated), and various passive elements. The calibration curve of the admittance from the sweat VIA sensor versus the wireless readout ADC data of the sensing circuit was obtained using reference data obtained through a circuit simulation (LTspice, Analog Devices) and measurement of the output voltage of the sensing circuit at a fixed admittance of the sweat VIA sensor.

### Fabrication of the sweat VIA-based epifluidic wireless patch

For the on-body study, the thickness of the sweat VIA channel was reduced from 1 mm to 350 μm to reduce the volume of the VIA channel and, accordingly, increase the spike frequency. The nanomesh interconnects electrodes of the sweat VIA sensor were connected to the wireless sensor system with an Au wire (diameter of 100 μm, Alfa Aesar). The sweat VIA sensor showed spike frequencies of ~0.015 Hz and 0.03 Hz at flow rates of 0.5 μL/min and 1 μL/min, respectively. The whole epifluidic patch, including the sweat VIA sensor, was attached to the skin using a double-sided biomedical adhesive containing a hole with a diameter of 5 mm that was aligned with the VIA channel.

### On-body study

The experimental protocols for the human-participant trials carried out to obtain on-body measurements were approved by the Institutional Review Board of the Korea Institute of Science and Technology (IRB# 2020-028, 2021-034). All participants (ages 25–35, healthy male individuals) were informed of and agreed to the risks and benefits of the measurements. The skin was cleaned using alcohol-containing cotton before applying the skin-sensing platform. Two epifluidic patches were attached to the chest skin area to monitor and compare the epidermal perspiration dynamics from different skin locations. Commercial absorbent cotton pads (Salivette®, SARSTEDT) were also attached to the skin to compare the data from the patches with data from sweat that was manually collected and analyzed using laboratory equipment. Each participant performed a sequence of stationary cycling exercises with various load levels for 90 min and then rested for 10 min. Once the epifluidic patches were attached, they were used throughout cycling and resting. In contrast, the cotton pads were replaced every 5 min, and the changes in their weights after each 5 min attachment were measured using a microbalance to calculate the sweat rate. The sweat samples absorbed by the cotton pads were collected using a centrifuge, and their impedance values were measured using a screen-printed electrode (DRP-250BT, Metrohm DropSens) and a potentiostat (VersaSTAT 3, Princeton Applied Research). The conductivity values were calculated using a calibration curve that correlated the impedance value with the solution conductivity obtained using standard solutions of known conductivity values (Supplementary Fig. S15).

## Supplementary information

Supplementary Information

## Data Availability

Data were available in the main article and supporting materials.
