# Supplementary material for: An epifluidic electronic patch with spiking sweat clearance for event-driven perspiration monitoring
Source: Nat Commun. 2022 Nov 7;13:6705. doi: 10.1038/s41467-022-34442-y (PMC9640696; doi:10.1038/s41467-022-34442-y)
Supplement: Supplementary file 1 — Supplementary Information [file 41467_2022_34442_MOESM1_ESM.pdf]

Supplementary Information:

An epifluidic electronic patch with spiking sweat clearance for event-driven perspiration monitoring

by Sangha Kim‡, Seongjin Park‡, Jina Choi‡, Wonseop Hwang‡, Sunho Kim, In-Suk Choi, Hyunjung Yi*, and Rhokyun Kwak*

‡These authors contributed equally to this work.
*Correspondence should be addressed to Hyunjung Yi (email: hjungyi@kist.re.kr) and Rhokyun Kwak (email: rhokyun@hanyang.ac.kr).

Inventory of Supplementary Information:

Supplementary Methods

Supplementary Discussion

Supplementary Figures S1 to S15

Supplementary References

Supplementary Methods

Characterizations: The morphology and the elemental mapping of the nanomesh electrodes were characterized using a scanning electron microscope (Nova NanoSEM 200, FEI) equipped with an energy-dispersive spectroscope (EDS) (Inspect F, FEI). The electrochemical impedance spectra of samples of AgNW/CNT and Ep-Au/AgNW/CNT in solutions of 100 mM NaCl were measured using an impedance analyzer (VersaSTAT 3, Princeton Applied Research) at an AC voltage of 30 mV. The electrochemical impedance spectra of various solutions, NaCl (20 mM, 50 mM) and NaCl (20 mM) with additional components such as KCl (20 mM), urea (30 mM), serine (150 mM), and glycine (150 mM) were also measured using the same impedance analyzer at an AC voltage of 30 mV and screen-printed electrodes (DRP-250BT, Metrohm DropSens).

Contact angles made by deionized water with a PDMS sponge and CNT-coated PDMS sponge were characterized by using a contact angle goniometer (Phoenix-MT(A), Surface Electro Optics, Korea). To measure the contact angles, the two sponges were made to have the same dimensions, namely $1.5 \times 1.5 \times 0.3$ cm^3, and a volume of 22 μL of the deionized water was dropped onto the surface of each sponge. Then, the static and/or dynamic motion of the droplet was recorded at 0.02 s intervals.

Measurement of the evaporation rate of the CNT-PDMS sponge: To quantify the rate of evaporation of water from the CNT-PDMS sponge, we first prepared two sponges with different sizes: $1.0\ \text{cm} \times 1.0\ \text{cm} \times 0.3\ \text{cm}$ and $1.5\ \text{cm} \times 1.5\ \text{cm} \times 0.3$ cm (Figure S5). After the sponge was fully wetted with 0.23 / 0.37 mL of deionized water, the change in the total mass was monitored by weighing the sponge using a high-precision balance every minute for 10 minutes. The evaporation rate (in volume per time) was calculated by dividing the mass loss per time with the density of the water, 0.997 g/mL at 25.5 °C. The experiment was performed in an isolated chamber, where the humidity and temperature were controlled to be 20 / 55% and 25.5 °C, respectively.

Long-term stability and reproducibility of the sweat VIA device: The performance of the sweat VIA devices was investigated under three different experimental conditions: i) 24-hour long-term stability test at a flow rate of 0.5 μL/min (< evaporation rate of the CNT-PDMS sponge), ii) 24-hour long-term failure test at a flow rate of 1 μL/min (> evaporation rate of the sponge), and iii) 8-hour reproducibility test with three devices at a flow rate of 0.5 μL/min. Here, we used a sweat VIA sensor with VIA channel of 0.35 mm height and a sponge with dimensions of 1.5 cm × 1.5 cm × 0.3 cm (the same dimensions with those used for the on-body test, Figure 6). The test solution was fixed as 100 mM NaCl solution, and the environment was 25.5 °C and 55% relative humidity.

Effect of the solution composition on the peak admittance and frequency of spike signals: The spike signals of the sweat VIA for each solution of 20 mM NaCl, 20 mM NaCl + 20 mM KCl, 20 mM NaCl + 30 mM urea, 20 mM NaCl + 150 mM serine, 20 mM NaCl + 150 mM glycine, and 50 mM NaCl were monitored by providing each solution to the VIA channel using a syringe pump at a fixed flow rate of 0.5 μL/min. The admittance of the sweat VIA was measured at a frequency of 50 kHz and 0.01 V using a potentiostat (Palm Sense). Each solution was measured after replacing the VIA channel with the test solution.

Self-heating effect of the sensor system during long-term operation: the self-heating effect was investigated at two different temperature conditions, i.e., room temperature (26 °C) and skin temperature (33 °C), since the bottom surface of the sensor platform is in contact with the chest. The skin temperature condition was emulated by placing the sensor system on a hot plate with the surface

temperature at 33 °C. Thermal images of the bottom surface of the sensor being operated for 8 h were obtained every hour using a thermal image camera (Fluke Ti95) with the emissivity set as 0.95, the emissivity of polyimide, the substrate material of the FPCB[1-3].

Supplementary Discussion

1. Theoretical analysis of the sweat VIA sensor

1.1. Correlation between sweat rate and spike frequency

The design parameters of the sweat VIA sensor are shown in **Figure 1a**. For the sweat VIA sensor, we estimated the time required to fill the VIA channel by using several equations, one of them being

$$Q^{''}A_{Inlet}t = \frac{1}{3}\pi h(t)(3r_0^2 + 3r_0 h(t)\cot\beta + (h(t)\cot\beta)^2),$$

where Q'' is the sweat rate per unit area of the skin at the inlet, A_{Inlet} is the inlet area of the VIA channel, and $h(t)$ id the sweat height at time t. r_o is the inlet radius of the channel and β is the wall angle of the channel. When the sweat fills the whole channel, if the design of the sweat VIA sensor is determined, this equation can be reformulated as

$$Q^{''}T = \frac{1}{3A_{Inlet}}\pi h_0(3r_0^2 + 3r_0 h_0\cot\beta + (h_0\cot\beta)^2) = constant\ A,$$

where T is the spike period and h_o is the height of the sweat when the sweat is in contact with the top of the channel, and r_o, h_o, β and A_{Inlet} are in this case each constant. As a result, the higher the sweat rate, the higher the spike frequency f (= $1/T$), with these data points able to be fit by the line defined by the equation $f = Q^{''}/A$ (red line in Fig. 3e).

However, at a high flow rate, the sweat clearance time would not be negligible, and the sweat collection time would become too short. Therefore, in this case, the correlation equation must be adjusted by considering the clearance time. Simply, with the assumption of a constant clearance time (B), the spike period T would be represented as the sum of the sweat collection and clearance times,

$$T = A/Q^{''} + B,$$

resulting in the non-linear equation $f = Q^{''}/(A + BQ^{''})$ (blue line in Fig. 3e) to fit the data.

1.2. Change of the electric conductance during the sweat collection and clearance processes

The relationship between the measured electric conductance, G, and the area of contact between the sweat and nanomesh electrode, $S(t)$, may be described using the equation

$$G = i/V = \left(\sum c_i\lambda_i \oiint_{S(t)} E\cdot dS\right)/V,$$

where i is current, V is voltage, c_i is the ion concentration of species i (e.g., Na^+,Cl^-), λ_i is the ion mobility of species i and E is the electric field generated by electrodes. During the course of a sweat collection, $S(t)$ would increase, and as a result so would the electric conductance —whereas during the course of a sweat clearance process, they would both decrease.

1.3. Correlation between admittance and sweat conductivity

The admittance of the filled VIA channel can provide information about sweat conductance. For sweat coming into contact with the top of the VIA channel, i.e., with the sweat height *h(t)* increased to h_o, *S(t)* and *G* would show their maximum values. For a channel fully filled with sweat, from Gauss's law, the conductance *G* would be

$$G = \frac{\sum c_i \lambda_i}{\varepsilon} \frac{q}{v} = \frac{\sum c_i \lambda_i}{\varepsilon} C,$$

for a closed surface *S* containing charge *q*, where *q/v* is the capacitance *C*, and ε is the permittivity of the electrolyte. Here the capacitance would be a constant due to the fixed geometry and material of the nanomesh electrode in the sweat VIA sensor. Therefore, the admittance (S) of the fully filled VIA channel would also be proportional to the ionic conductivity of the sweat in the VIA channel.

Supplementary Figures

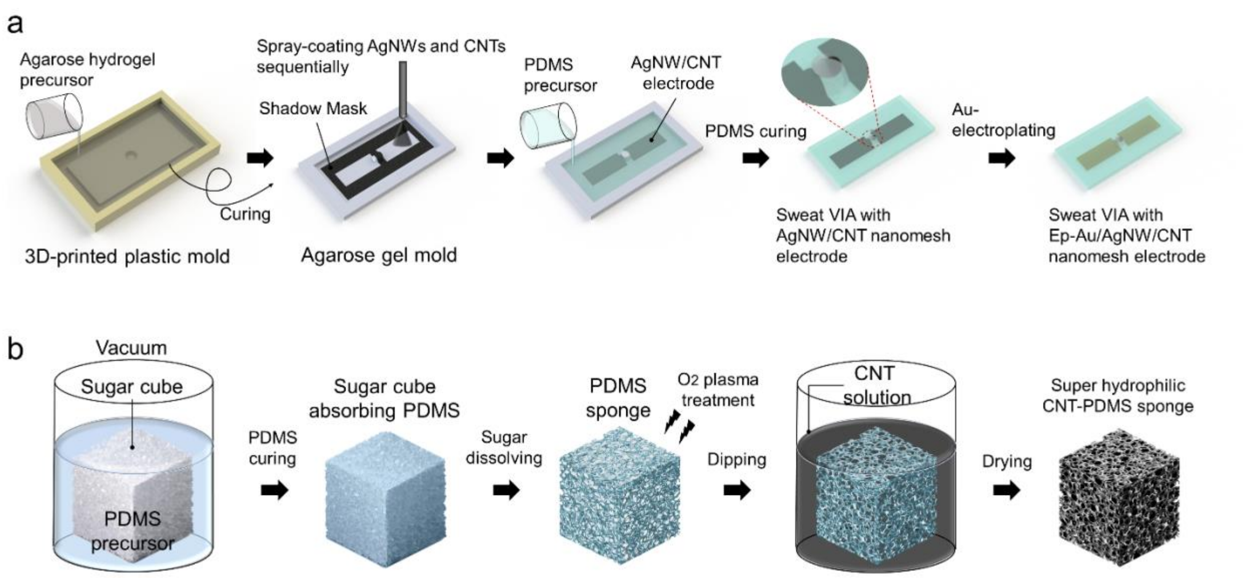

Figure S1. Fabrication of the sweat VIA sensor components. a. Fabrication of the sweat VIA sensor with nanomesh electrodes. Agarose hydrogel precursor was molded using a 3D-printed plastic mold engraved with a truncated cone. AgNW and CNT solutions were sequentially spray-coated through a pre-patterned shadow mask onto the cured agarose gel mold. Then, a PDMS-based sweat VIA channel was molded from the agarose gel mold, after which patterned AgNWs and CNTs were transferred to the PDMS. Then, an Au layer was electroplated onto the transferred AgNW/CNT nanomesh electrodes on the PDMS surface. **b.** Fabrication of the super-hydrophilic CNT-PDMS sponge. A sugar cube was immersed in a PDMS precursor under vacuum conditions. The sugar cube absorbed the PDMS precursor, and the resulting composite was cured, and then dissolved in warm water to dissolve the sugar component and produce a PDMS sponge. Finally, the sponge was treated with O_2 plasma and then dipped into a CNT solution to produce a CNT-PDMS sponge.

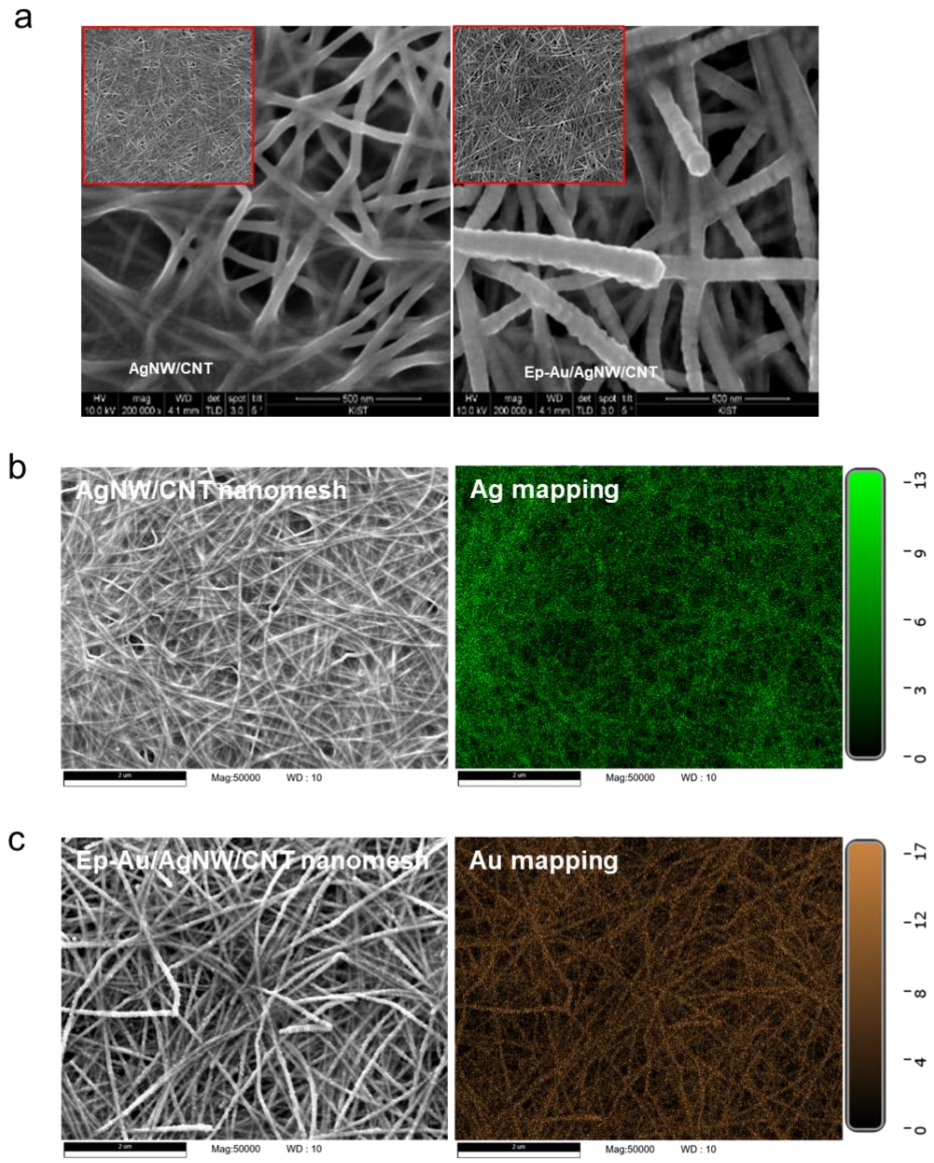

Figure S2. Characterizations of the nanomesh electrodes. a. Scanning electron micrographs of the AgNW/CNT (left) and the Ep-Au/AgNW/CNT (right) electrodes. **b-c.** SEM-EDS mapping images of Ag and Au within (b) the AgNW/CNT nanomesh and (c) Ep-Au/AgNW/CNT nanomesh.

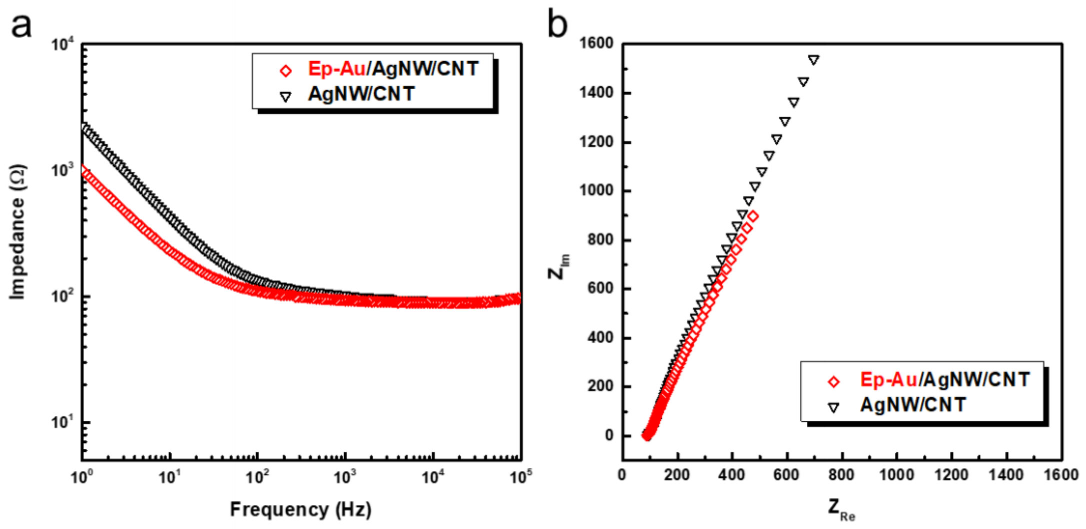

Figure S3. EIS plots of the Ep-Au/AgNW/CNT and AgNW/CNT nanomesh electrodes each in a 100 mM NaCl solution. a. Bode plots. **b.** Nyquist plots. An AC voltage of 30 mV (vs. Ag/AgCl (3 M NaCl saturated)) was applied in each case.

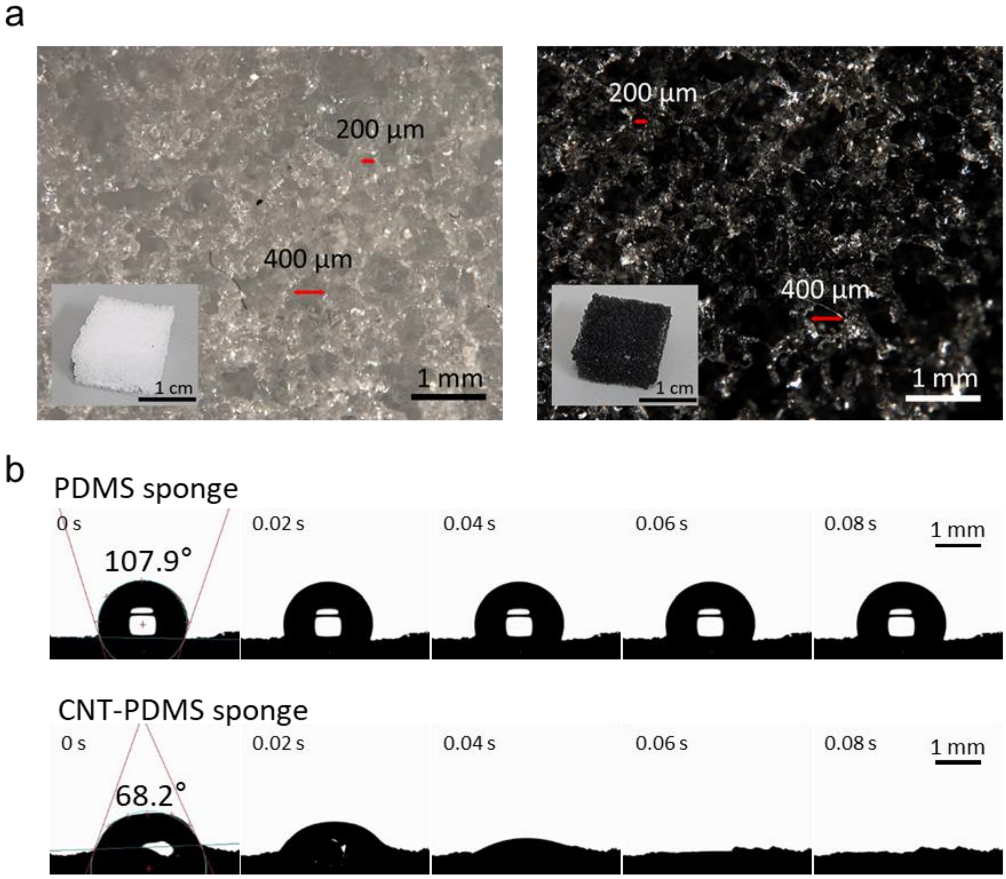

Figure S4. Contact angles for the PDMS sponge and CNT-coated PDMS sponge. a. Microscopy images of a PDMS sponge (before coating with CNTs) and CNT-PDMS sponge. The two sponges showed essentially identical pore sizes. **b.** Contact angles of DI water on the two sponges. The PDMS sponge showed a contact angle of 107.9° (hydrophobic), whereas we were unable to determine the contact angle on the CNT-PDMS sponge determined because the droplet absorbed spontaneously onto the entire extent of the sponge within 0.05 s.

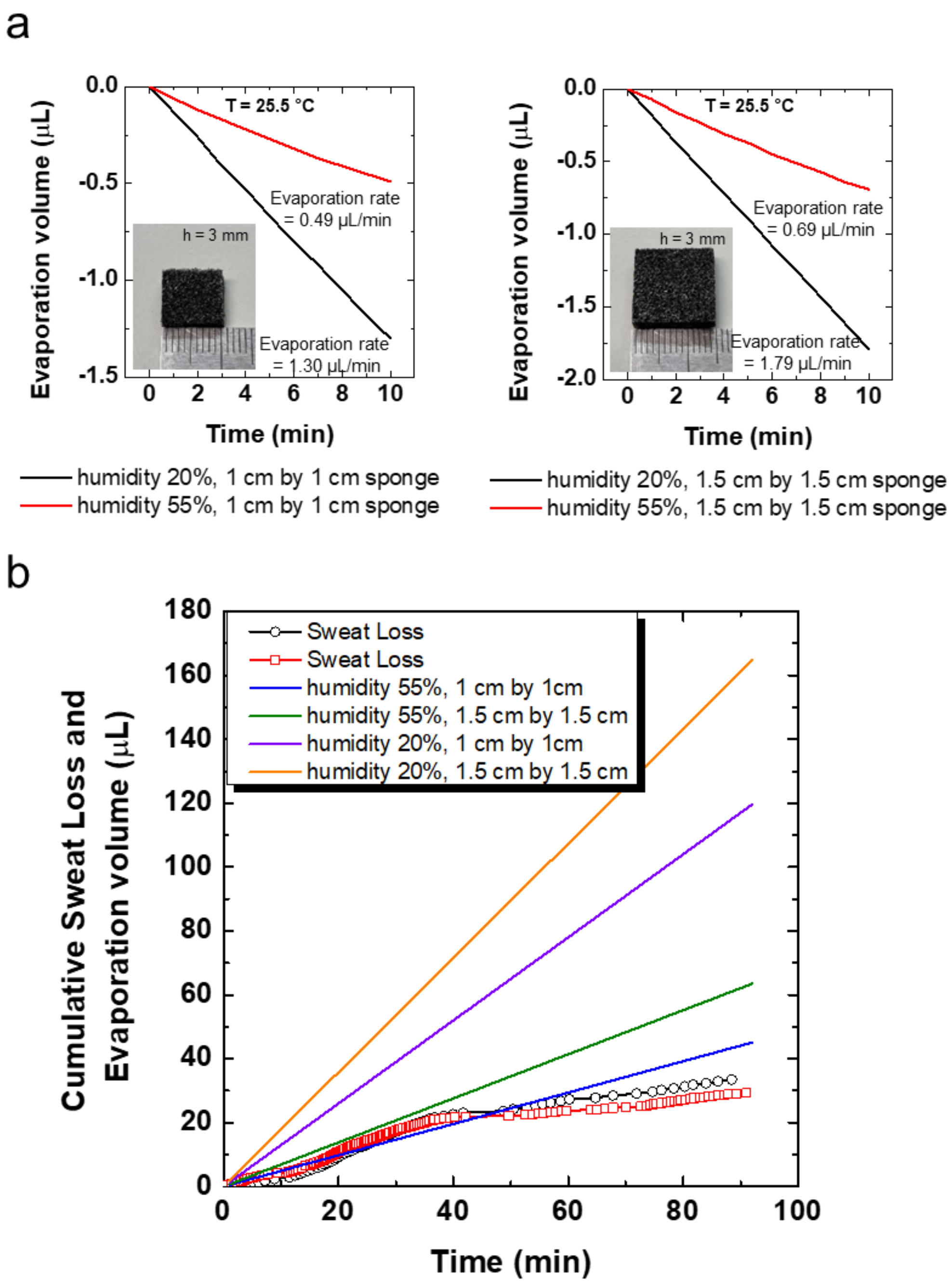

Figure S5. Rates of evaporation of DI water from the CNT-coated PDMS sponge under various conditions. a. Rates of evaporation of DI water from two sizes of the CNT-coated PDMS sponge (1 x 1 x 0.3 and 1.5 x 1.5 x 0.3 cm^3) each at 25.5 °C with relative humidity values of 20% and 55%. **b.** Comparison of evaporation volumes of the four cases and the cumulative sweat loss from the on-body measurement (Figure 5f). The highest evaporation rate was observed for the larger sponge at the lower relative humidity. In all cases, the cumulative evaporation volumes were greater than the cumulative sweat loss from the on-body test; thus, the sweat VIA sensor can be operated for a long period of time.

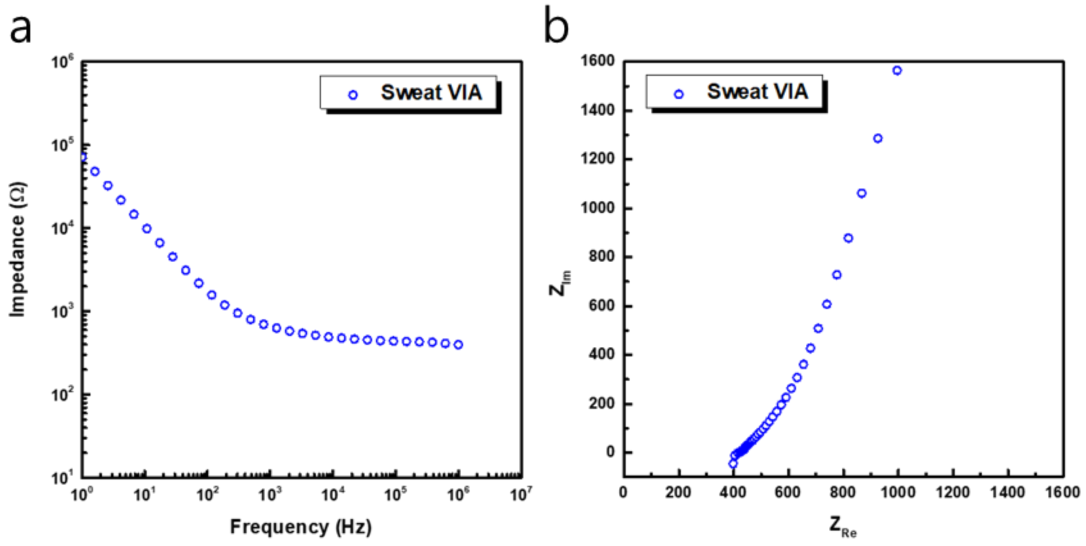

Figure S6. EIS plots of the sweat VIA sensor filled with a solution of 100 mM NaCl. a. Bode plot. **b.** Nyquist plot. Data were collected using an AC voltage with an amplitude of 30 mV.

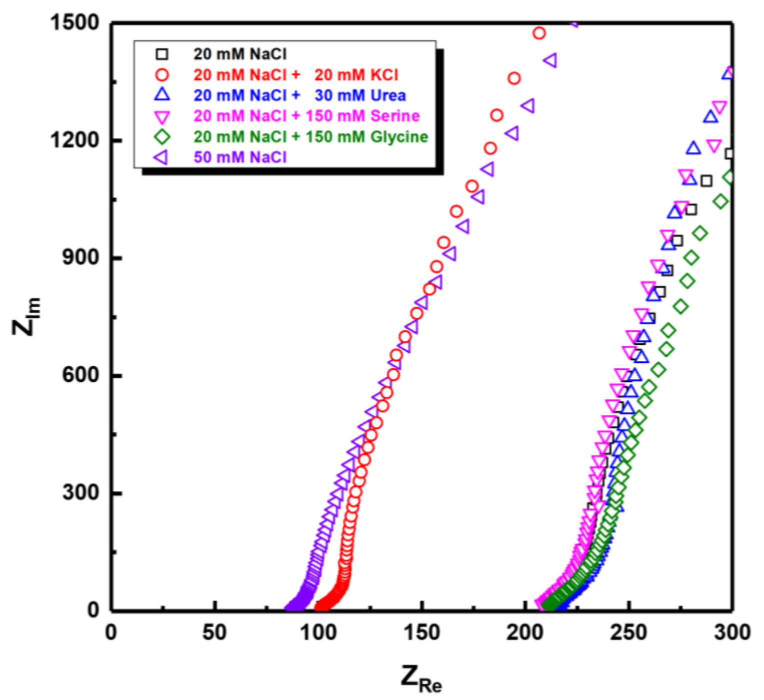

Figure S7. Nyquist plots of various solutions. NaCl (20 mM, 50 mM) and NaCl (20 mM) with additional components such as KCl (20 mM), urea (30 mM), serine (150 mM), and glycine (150 mM) were characterized.

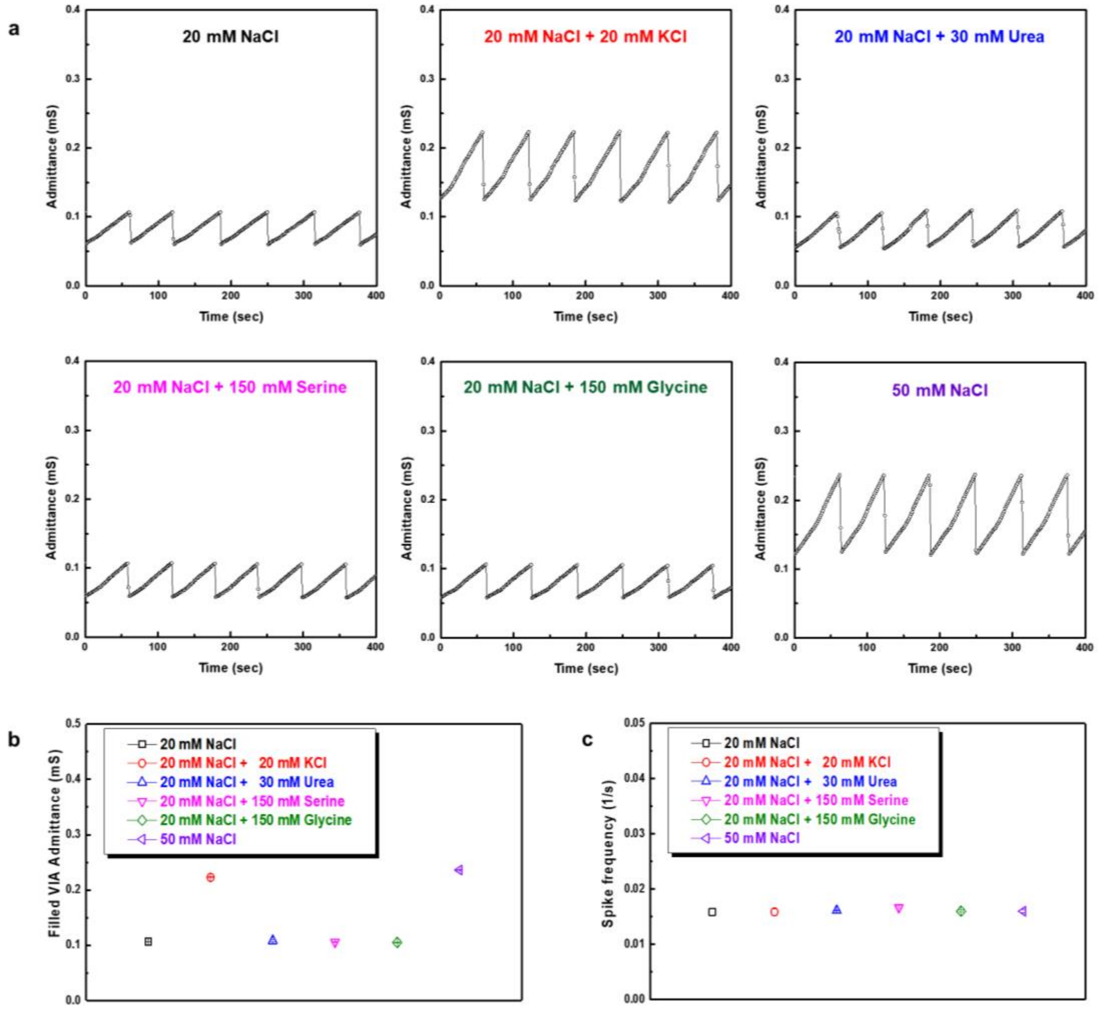

Figure S8. Effect of the solution composition on the peak admittance and frequency of spike signals. **a.** Monitoring of the admittance of the sweat VIA sensor with solutions of various compositions. The flow rate of the solutions was fixed at 0.5 μL/min. **b.** Maximum admittance and **c.** frequency of the admittance spike signals for the solutions. The admittance and frequency were averaged from the five spikes of each solution.

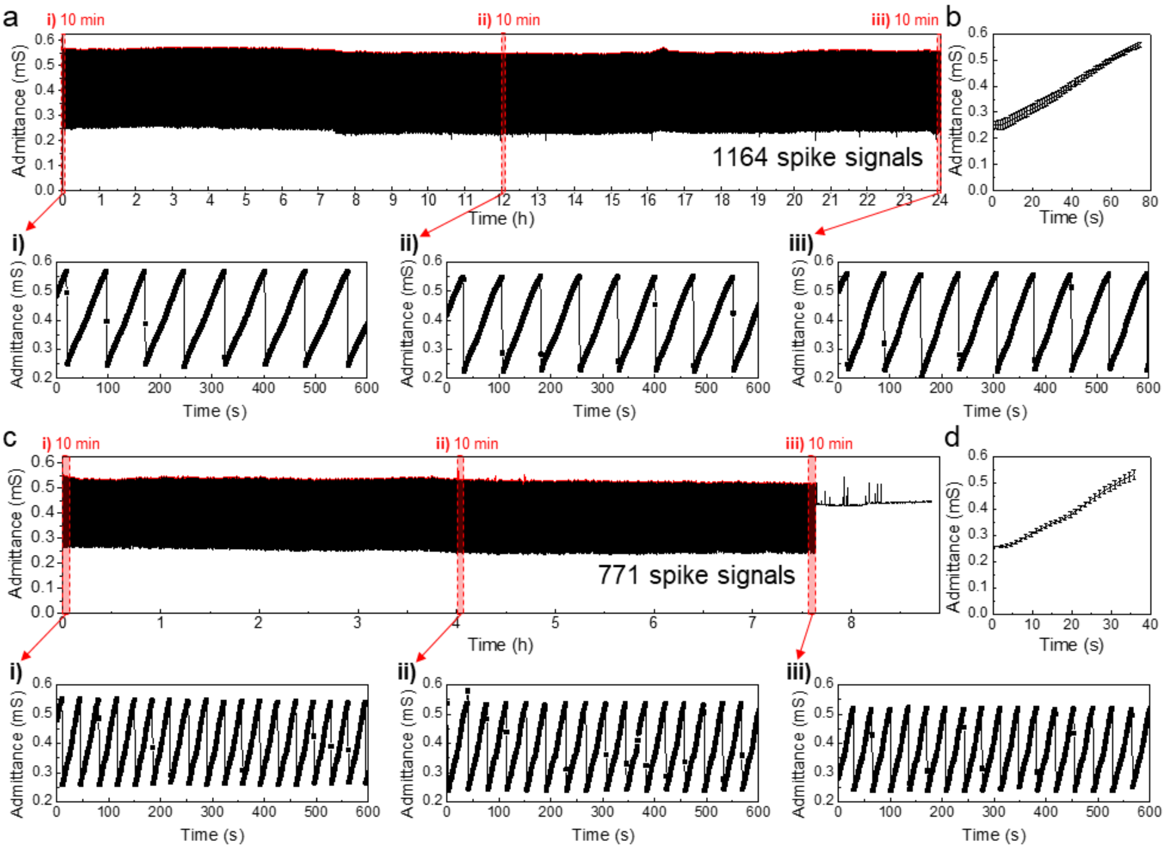

Figure S9. 24-hour long-term stability test. Long-term stability test results with **a.** 0.5 µL/min and **c.** 1 µL/min flow rates. Enlarged curves are presented at the (i) beginning (0 h), (ii) middle (4 or 12 h), and (iii) final (24 or 7.6 h) with 10-minute intervals. Averages of all spike signals obtained from the stability test with **b.** 0.5 µL/min and **d.** 1 µL/min flow rate. The amplitude and period of the averaged spike signal are 0.308 mS and 74.4 s at 0.5 µL/min (RSD 1.06%), 0.275 mS and 35.8 s at 1 µL/min (RSD 3.25%), respectively. At 1 µL/min, the volume of the residual sweat within the sponge at the device failure time point (27,514 s or ~7.6 h) is ~ 0.14 mL, which is calculated from 0.31 µL/min (1 µL/min (flow rate) - 0.69 µL/min (evaporation rate, Figure S5) × 27,514 s. This value is about 38% of the sponge's absorption capacity (0.37 mL, Figure S5).

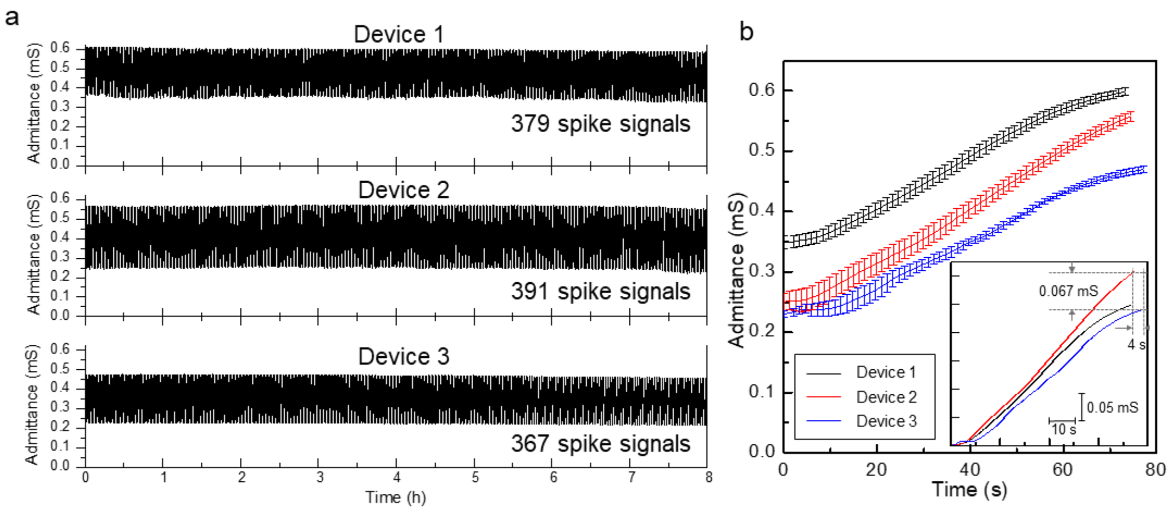

Figure S10. 8-hour reproducibility test. a. Eight-hour reproducibility test with three different devices at a flow rate of 0.5 μL/min. **b.** Averaged admittance curves of each of the three different devices. The amplitudes and periods of the averaged spike signals are 0.249 mS/ 72.2 s (RSD 1.06%), 0.304 mS/ 74.4 s (RSD 1.47%) and 0.240 mS / 77.2 s (RSD 1.17%), respectively. The slight variations were ascribed to the fabrication tolerances that induced variations in the channel volume (e.g., channel height) and the sponge thickness.

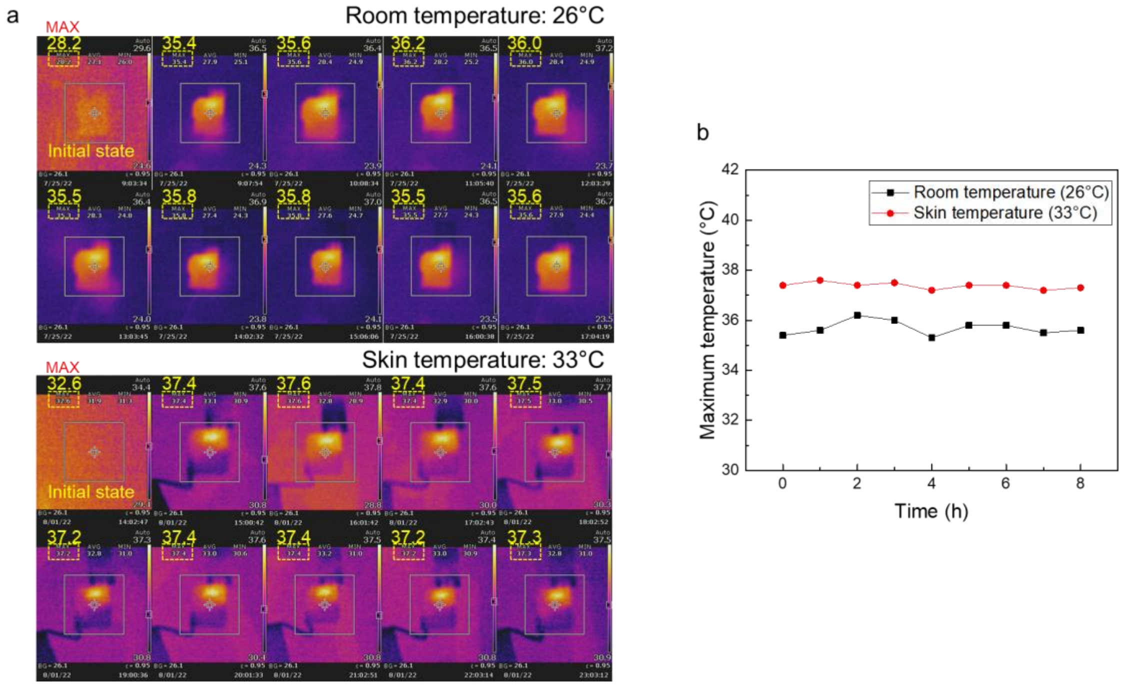

Figure S11. Self-heating effect of the sensor system during long-term operation a. Thermal camera images of the bottom surface of the sensor system being operated for 8 h at room temperature (26 °C) and skin temperature (33 °C) conditions. **b.** The maximum temperature level of the bottom surface of the sensor system at room temperature and skin temperature conditions.

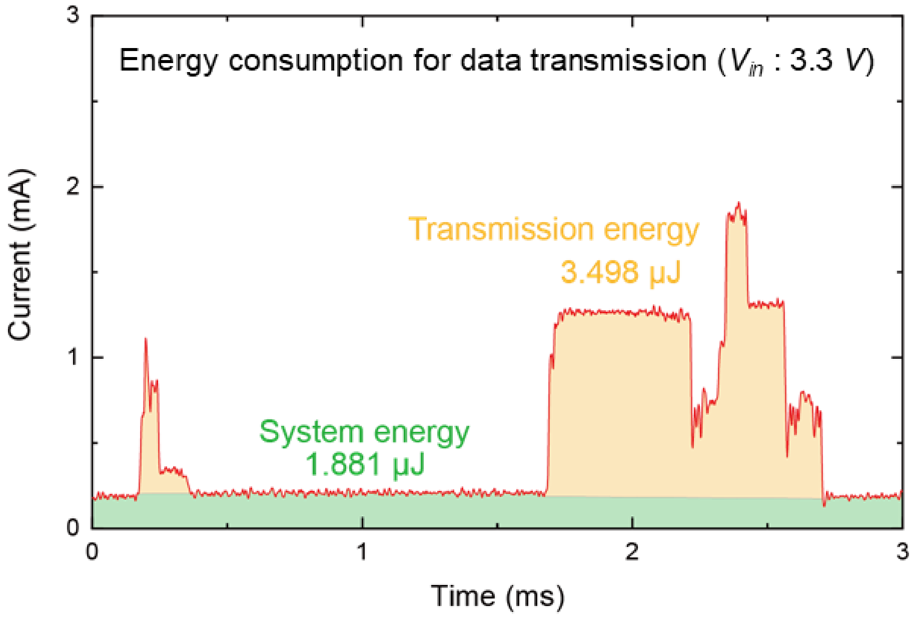

Figure S12. **Power consumption levels for data transmission and system operation of the sweat VIA-based epifluidic wireless patch**. The energy level was calculated based on the measured current level for 3 ms during data communication.

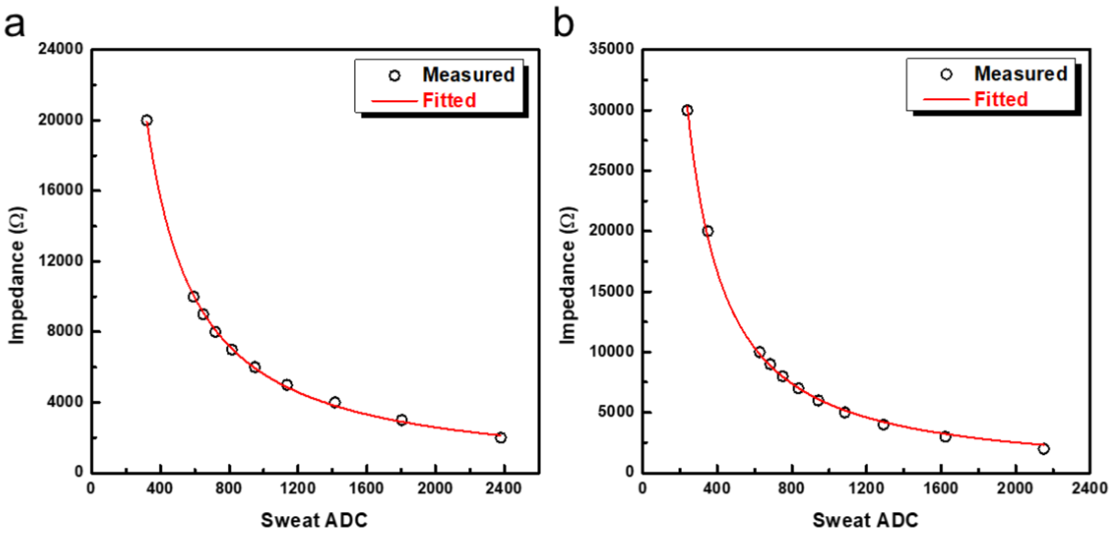

Figure S13. Calibration curves of the impedance from the sweat VIA sensor versus the wireless readout of the sweat VIA-based epifluidic patch used for monitoring of perspiration dynamics of chest areas. a. The epifluidic wireless patch attached at the *R* position. **b**. The epifluidic wireless patch attached at the *C* position. The measured data points were fit using the equation $y = A \times (1 + x)^B$, where x and y denote ADC data and impedance, respectively, and A and B are coefficients whose values were determined by carrying out repetitive fitting plot simulations. A:1.3×10^7, B:-1.1. for (**a**) and A:1.9×10^7, B:-1.2 for (**b**).

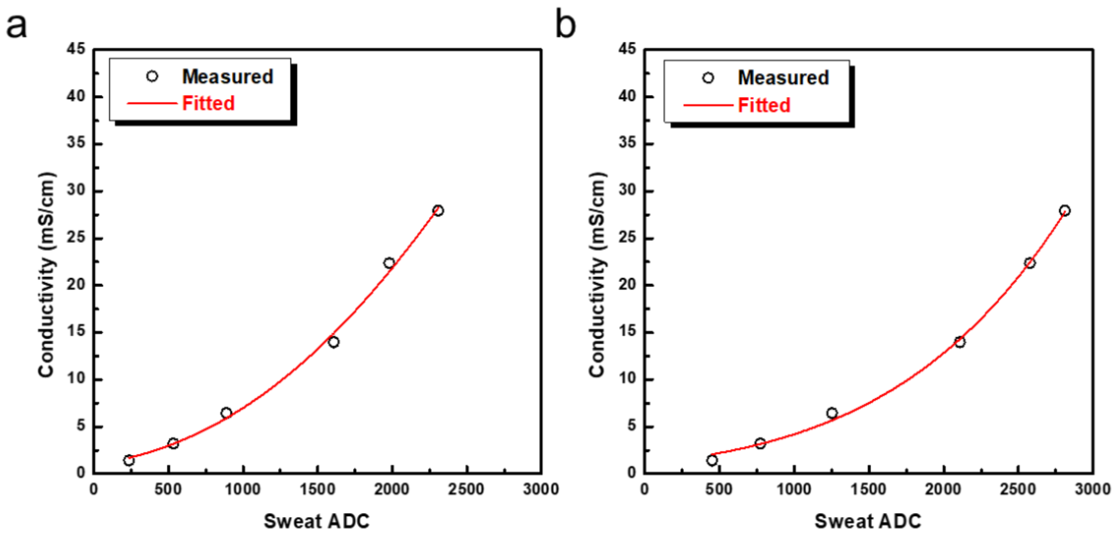

Figure S14. Calibration curves of the sweat conductivity versus the wireless readouts of the sweat VIA-based epifluidic patch. a. The epifluidic wireless patch attached at the *R* position. **b**. The epifluidic wireless patch attached at the *C* position. The measured data points were fit using the equation $y = A \times e^{(B+\frac{C}{x+D})}$, where x and y denote ADC data and conductivity, respectively, and A, B, C, and D are coefficient values. A:1.0, B:7.2, C:-1.9$\times 10^4$, D:2.6 $\times 10^3$ for (**a**) and A:1.8 $\times 10^6$, B:0, C:-1.4$\times 10^5$, D:9.6 $\times 10^3$ for (**b**).

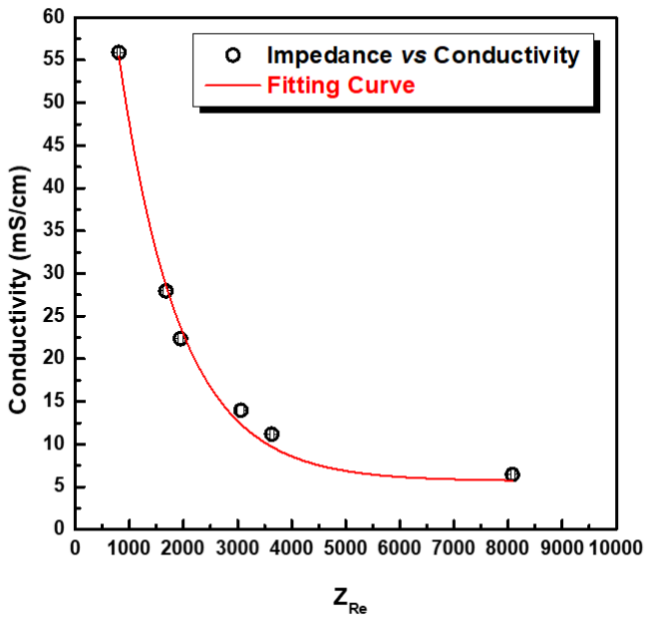

Figure S15. A calibration curve of the conductivity versus the solution impedance (Z_{Re}) of the sweat collected using absorption pads. The measured data points were fit using the equation $y = A + B \times e^{(\frac{-x+C}{D})}$, where x and y denote impedance and admittance, respectively, and A, B, C, and D are coefficient values. A:5.7 B:51.7, C:7.6$\times 10^2$, D:1.1 $\times 10^3$.

Supplementary References

1. Singh, D.K., Donovan, S., Pardyjak, E.R. & Garrett, T.J. A differential emissivity imaging technique for measuring hydrometeor mass and type. *Atmos. Meas. Tech.* **14**, 6973-6990 (2021).
2. Choi, J.-H. & Loftness, V. Investigation of human body skin temperatures as a bio-signal to indicate overall thermal sensations. *Building and Environment* **58**, 258-269 (2012).
3. Costa, C.M.A. et al. Daily rhythm of skin temperature of women evaluated by infrared thermal imaging. *Journal of Thermal Biology* **72**, 1-9 (2018).
